# A Lytic Bacteriophage as a Potential Biocontrol Agent Against Avian Enterotoxigenic *Escherichia coli* Associated With Colibacillosis

**DOI:** 10.1155/cjid/6665750

**Published:** 2025-12-30

**Authors:** Arina Sasoon, Farhad Nikkhahi, Niloofar Kiaheyrati, Amir Javadi, Amir Peymani, Anita Fard Sanei, Fatemeh Fardsanei

**Affiliations:** ^1^ Medical Microbiology Research Center, Qazvin University of Medical Sciences, Qazvin, Iran, qums.ac.ir; ^2^ Department of Community Medicine, School of Medicine, Qazvin University of Medical Sciences, Qazvin, Iran, qums.ac.ir; ^3^ Ramsar Campus, Mazandaran University of Medical Sciences, Ramsar, Iran, mazums.ac.ir; ^4^ Dietrich School of Arts and Sciences, University of Pittsburgh, Pittsburgh, Pennsylvania, USA, pitt.edu

**Keywords:** antibiotic resistance, bacteriophage, biofilm, enterotoxigenic *Escherichia coli*

## Abstract

**Introduction:**

Diarrheal diseases remain a major public health concern, causing over two million deaths annually in developing countries. Diarrheagenic *Escherichia coli*, particularly enterotoxigenic strains, are a leading cause of gastrointestinal infections in humans and animals. Poultry can act as a reservoir for pathogenic *E. coli*, facilitating transmission to humans via contaminated food or water. This study aimed to isolate and characterize bacteriophages targeting *E. coli* pathotypes from poultry, with the goal of controlling gastrointestinal infections and reducing the risk of zoonotic transmission.

**Methods:**

ETEC strains were isolated from poultry with colibacillosis and confirmed by LT and ST toxin genes. Their antibiotic resistance and biofilm formation were evaluated. Bacteriophages were isolated from wastewater, and their host range, morphology, growth characteristics, lytic activity, and stability under different temperatures and pH were assessed.

**Result:**

The isolated ETEC strain exhibited a multidrug‐resistant phenotype and strong biofilm formation. The bacteriophage was highly specific for this strain, showing no lysis of other bacteria, and was classified as belonging to the Autographiviridae family by TEM analysis. One‐step growth experiments revealed a latent period of 0–10 min and a burst size of 93 PFU/cell. Lytic activity was effective at MOIs of 10 and 100, and the phage remained stable under typical environmental and physiological conditions, suggesting its potential applicability in poultry.

**Conclusion:**

The isolated phage demonstrates high specificity, lytic activity, and stability, indicating its potential as an alternative or adjunct to antibiotics for controlling MDR ETEC in poultry. Further studies are warranted to evaluate its safety and efficacy under in vivo conditions.

## 1. Introduction

Diarrheal diseases constitute a significant global public health concern, accounting for approximately two million deaths annually in developing countries. Among the various bacterial pathogens, diarrheagenic *E. coli* (DEC) strains are recognized as the predominant causative agents of diarrhea, especially in developing nations. According to their virulence factors, DEC is categorized into six distinct groups: enterotoxigenic *E. coli* (ETEC), enteropathogenic *E. coli* (EPEC), enterohemorrhagic *E. coli* (EHEC), enteroinvasive *E. coli* (EIEC), enteroaggregative *E. coli* (EAEC), and diffusely adherent *E. coli* (DAEC) [1, 2].

ETEC is the most prevalent foodborne and zoonotic pathogen causing diarrhea in humans and animals, potentially leading to death in severe cases. As this bacterium is common in both animal and human bodies, it is disseminated to the environment and humans mostly through livestock [3]. ETEC is also the primary etiology of travelers’ diarrhea, which is prevalent in low‐income regions of the globe. Gastrointestinal disturbances are diagnosed in approximately one‐third of all travelers’ diarrhea patients who seek medical care. ETEC generates two types of virulence factors: colonization factors (CFs) and enterotoxins. Enterotoxins attach to certain receptors on intestinal epithelial cells, causing diarrhea, whereas CFs aid in the bacteria’s adhesion to these cells [4]. ETEC is characterized by its ability to produce heat‐labile (LT) and heat‐stable (ST) enterotoxins. ETEC induces watery diarrhea, which may vary from mild, self‐limiting conditions to severe illnesses [5]. Although ETEC infections in travelers are typically self‐limited, antibiotics can hasten symptom resolution. However, recent studies have raised concerns regarding the acquisition and subsequent asymptomatic carriage of multidrug‐resistant (MDR) *Enterobacteriaceae*, including *E. coli*, among travelers. This phenomenon is largely attributed to the selective pressure imposed by antibiotics used for either therapeutic or prophylactic purposes [6].

The indiscriminate use of antibiotics has significantly contributed to the rise of MDR bacteria. Therefore, the rise in infection severity and treatment failure requires safe and efficient alternatives. The phages are garnering attention as a potential alternative to antibiotics due to their ability to circumvent the resistance developed to antibiotics through various mechanisms and their low risk of staying in the intestine [7]. In contrast to antibiotics, which alter the balance of microbial flora and affect a wide variety of bacteria in the body because of their broad spectrum, phages are entirely host‐specific. Bacteriophages are viruses that infect and lyse bacterial cells. Furthermore, bacteriophages are self‐replicating agents that are nonhazardous and increase in number as they eliminate target bacteria [8]. Another advantage of bacteriophages is their ability to penetrate biofilm structures [9]. Bacteriophages are not only highly specific to their bacterial hosts but also possess enzymatic mechanisms that enhance their ability to eradicate biofilm‐associated infections [10]. Many phages produce depolymerases, polysaccharide‐degrading hydrolases, and endolysins that can disrupt the extracellular polymeric substances of biofilms, allowing the phage particles to access and lyse the embedded bacterial cells. This enzymatic activity significantly contributes to their potential as alternative or complementary agents to antibiotics, especially against MDR and biofilm‐forming pathogens [11, 12].

The present study aimed to isolate and comprehensively characterize a bacteriophage specifically active against enterotoxigenic *Escherichia coli* (ETEC) and to evaluate its antibacterial efficacy and potential application as a biological control agent.

## 2. Materials and Methods

### 2.1. Ethical Approval

The methods employed in this research adhered to the guidelines set forth by the Ethics Committee of Qazvin University of Medical Sciences, as evidenced by Approval Number IR.QUMS.REC.1401.330.

### 2.2. Sampling and Bacterial Isolation

A MDR and strong biofilm‐forming *enterotoxigenic Escherichia coli* (ETEC) strain, previously isolated from poultry, was selected for bacteriophage evaluation in this study. The strain was chosen based on its virulence characteristics, including resistance to commonly used antibiotics and the ability to produce robust biofilms [13].

### 2.3. Pathotyping of ETEC Isolates

Pathotyping of *Escherichia coli* isolates was carried out by polymerase chain reaction (PCR) targeting the genes encoding LT and ST enterotoxins, which are the main virulence determinants of ETEC. For LT gene amplification, the primer pair LT‐F (5′‐GGC​GAC​AGA​TTA​TAC​CGT​GC‐3′) and LT‐R (5′‐CGG​TCT​CTA​TAT​TCC​CTG​TT‐3′) was used, whereas for ST gene amplification, primer pair ST‐F (5′‐AGG​AAC​GTA​CAT​CAT​TGC​CC‐3′) and ST‐R (5′‐CAA​AGC​ATG​CTC​CAG​CAC​TA‐3′) was employed. The primers used in this study were synthesized by Metabion (Germany). Lyophilized primers were reconstituted according to the manufacturer’s instructions to prepare stock solutions [14].

### 2.4. Antibiotic Susceptibility Testing

An antibiotic susceptibility test for the enterotoxigenic *Escherichia coli* strain was performed using the Kirby–Bauer disk diffusion method, according to the Clinical and Laboratory Standards Institute (CLSI) 2023 guidelines. Various antibacterial disks (Thermo Fisher Scientific, USA), including cefepime (20 μg), cefotaxime (30 μg), ceftazidime (30 μg), cefoxitin (5 μg), neomycin (30 μg), ciprofloxacin (5 μg), lincospectin (30 μg), tylosin (40 μg), danofloxacin (40 μg), tetracycline (30 μg), enrofloxacin (10 μg), doxycycline (30 μg), trimethoprim/sulfamethoxazole (25 μg), imipenem (10 μg), ampicillin (30 μg), florfenicol (30 μg), and levofloxacin (5 μg), were selected and tested. In addition, colistin sensitivity was also determined using the colistin broth dilution elution (CBDE) method according to CLSI guidelines [15].

### 2.5. Biofilm Formation Assay

The formation of biofilms was studied using a 96‐well microtiter plate technique. Bacterial cultures were grown in a tryptic soy broth (TSB) medium containing 5% sucrose and then adjusted to a standard concentration (0.5 McFarland) before being added to each microplate well. The plate was then incubated at 37°C for 18–24 h. *Pseudomonas aeruginosa* (ATCC 27853) and uncultured TSB medium (Merck, Germany) were the positive and negative controls, respectively. Following three quick washes with phosphate‐buffered saline (PBS), and cell fixation with 95% methanol solution, the plate was stained with 200 μL of 1% crystal violet (CV) for 15 min. The dye was taken out, and the wells were rinsed three times with PBS. Subsequently, the biofilms formed were dissolved with 200 μL of 33% acetic acid for 30 min. A microtiter plate reader (BioTek, Epoch, USA) was used to measure the optical absorbance at 570 nm (OD570 and ODC570). Biofilms were categorized into four groups based on the ODc value: OD ≤ ODc (nonbiofilm formation), ODc < OD ≤ 2 ODc (weak biofilm formation), 2 ODc < OD ≤ 4 ODc (medium biofilm formation), and OD > 4 ODc (strong biofilm formation) [16].

### 2.6. Bacteriophage Isolation and Purification

**Figure 1:**
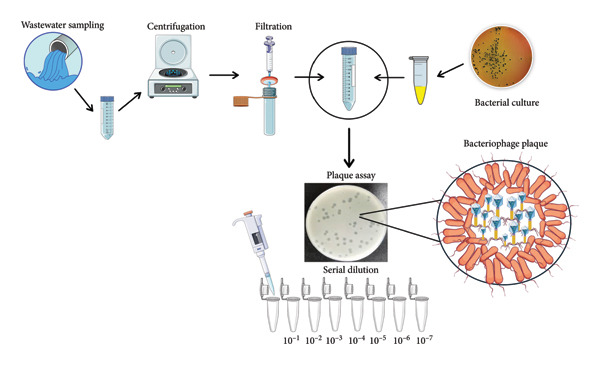
Schematic representation of the bacteriophage isolation process. Wastewater samples were collected, filtered, and enriched with *Escherichia coli*. Phages were subsequently detected and quantified using the double‐layer agar method.

To isolate the *Escherichia coli* phage, wastewater samples were collected from Velayat Hospitals in Qazvin Province, Imam Khomeini Hospital, and Children’s Medical Center in Tehran Province. Samples were continuously sent to the laboratory until the bacteriophage was isolated. These samples were kept at 4°C for 24 h to allow sedimentation. Subsequently, 15 mL of the sample was centrifuged in a Falcon tube for 10 min at 6000 rpm to allow the remaining coarse sediment to settle. The supernatant was passed through 0.22‐micron membrane filters. To enhance the presumed phage, 5 mL of the filtered phage solution was combined with 5 mL of TSB medium containing *Escherichia coli* at a concentration corresponding to 0.5 McFarland’s standard and incubated at 37°C for 24 h. Then, it was subjected to centrifugation and filtration once again. The presence of phages was confirmed using the double‐layer agar method. Briefly, 100 μL of the filtered lysate was mixed with 400 μL of TSB containing *E. coli* at 0.5 McFarland and incubated at 37°C for 10 min. This suspension was then combined with 4.5 mL of TSB containing 0.7% agar, previously melted and cooled to 50°C, and immediately poured onto the surface of TSA (Merck, Germany) plates containing 1.5% agar. Plates were allowed to solidify at room temperature for 10 min before further incubation. Plates were incubated at 37°C for 24 h. The following day, the presence of phage plaques was assessed by visual inspection [17] (Figure 1).

### 2.7. Phage Host Range

The sensitivity of bacterial strains to the isolated phage was assessed using the spot test. According to this method, a fresh culture of standard bacterial strains available in the laboratory was prepared in LB broth medium. Subsequently, 100 μL of each bacterial culture was mixed with 5 mL of 0.7% LB agar, cooled to 50°C, and thoroughly vortexed. The mixture was then poured onto the surface of 1.5% LB agar plates. Next, 10 μL of the phage suspension was spotted onto the center of the overlay agar without contacting the agar surface. The plates were incubated for 24 h at 37°C. The presence of a nongrowth plaque at the phage inoculation site is a sign of the sensitivity of the target bacteria to the phage, and the absence of a nongrowth plaque means that the phage has not been able to lyse that bacterium [18]. The strains used in this section were as follows:

1. *Klebsiella pneumoniae* ATCC 700603
2. *Escherichia coli* ATCC 25922
3. *Staphylococcus aureus* ATCC 225923
4. *Shigella flexneri* ATCC 12022
5. *Salmonella enterica* serovar *Typhimurium* ATCC 14028
6. *Enterococcus faecalis* ATCC 29212
7. *Enterobacter aerogenes* ATCC 13048 2.5

### 2.8. Transmission Electron Microscopy (TEM)

Bacteriophage morphology was analyzed using TEM following the protocol described by Taha et al. [19] at the Tehran Applied Pharmaceutical Research Center. First, a suspension with a high concentration of phage was prepared with repeated passages of phage in TSB medium. A drop of the phage suspension was placed on a copper grid coated with carbon. After 5 min, when the sample dried, the grid was stained with 2% uranyl acetate and observed with a microscope.

### 2.9. One‐Step Growth

A one‐step growth experiment was conducted to determine the latent time and burst size of the ETEC phage. Briefly, in this step, we prepared a suspension of 500 μL of host bacteria at a concentration of 10^8^ CFU/mL and 10^8^ PFU/mL phage particles with a multiplicity of infection (MOI) of 1. Then, it was incubated for 10 min at 37°C. After that, this solution was centrifuged in a refrigerated centrifuge at 4°C, 7000 rpm, and its supernatant was discarded, and the pellet was washed twice with TSB. Then, the pellet was added to 10 μL of TSB and incubated in a shaking incubator at 37°C, 160 rpm, 180 min. In the next step, latent time and burst size were determined by the DLA method at 5‐min intervals for at least 1 h [20].

### 2.10. Thermal and pH Stability of the Phage

For the pH stability assay, bacteriophage lysate was added to a series of tubes containing SM buffer adjusted to different pH values (3, 4, 5, 6, 7, 8, 9, and 10) for 10 h. For the thermal stability assay, aliquots of the lysate were incubated at various temperatures (4°C, 25°C, 37°C, 45°C, 55°C, 60°C, 65°C, and 70°C) for 1 h. Following treatment, the phage titer was determined using the double‐layer agar method [21]. All experiments were performed in triplicate, and each assay was independently repeated at least three times to ensure reproducibility of the results [17].

### 2.11. Determination of Optimal MOI

The optimal MOI is defined as the ratio of phage particles to host bacterial cells that results in the highest phage yield. The host bacterium was cultured in TSB, and the bacterial concentration (CFU/mL) was calculated using the following formula: CFU/mL = (number of colonies ∗ dilution factor)/volume plated. Similarly, the phage concentration (PFU/mL) was determined as follows: PFU/mL = (number of plaques ∗ dilution factor)/volume plated.

The MOI was obtained by dividing the number of infectious phage particles (PFUs) by the number of bacterial cells (CFUs). Phage suspensions were added to the bacterial culture (1.0 × 10^8^ CFU/mL) to achieve MOIs of 0.01, 0.1, 1, and 10, followed by incubation at 37°C with shaking at 180 rpm for 5 h. After incubation, the phage titers were determined using the double‐layer agar method, and the MOI producing the highest titer was considered the optimal MOI. All experiments were conducted in triplicate [22].

### 2.12. Statistical Analysis

SPSS software version 22 was used for conducting statistical analysis. All graphs were generated using GraphPad Prism. The data are presented as mean ± standard deviation (SD) from three independent experiments. The result was considered to have statistical significance if the *p*‐value was less than 0.05 (*p* < 0.05).

## 3. Results

### 3.1. Characterization of ETEC Isolate

An enterotoxigenic *Escherichia coli* (ETEC) strain was identified among the *E. coli* isolates obtained from poultry with colibacillosis, based on the presence of the LT and ST toxin genes.

### 3.2. Antibiotic Susceptibility Testing and Biofilm Formation Assay of ETEC Strain

**Figure 2:**
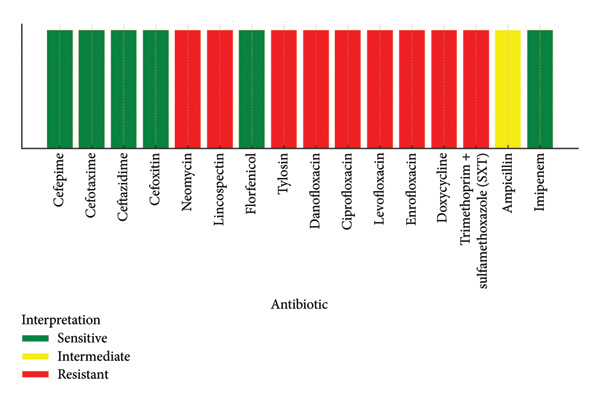
Antibiotic susceptibility profile of the ETEC strain isolated from poultry with colibacillosis.

Antimicrobial susceptibility testing revealed that the ETEC strain was resistant to neomycin, tylosin, danofloxacin, ciprofloxacin, levofloxacin, enrofloxacin, doxycycline, and trimethoprim–sulfamethoxazole. The ETEC isolate was found to be susceptible to colistin. The antimicrobial susceptibility profile of the *enterotoxigenic Escherichia coli* (ETEC) strain is presented in Figure 2. These findings indicate that the isolate possesses a MDR phenotype, particularly against aminoglycosides, macrolides, fluoroquinolones, and tetracyclines. The biofilm formation assay indicated that this strain possessed a strong ability to produce robust biofilms, reflecting its high virulence potential.

### 3.3. Phage Isolation

**Figure 3:**
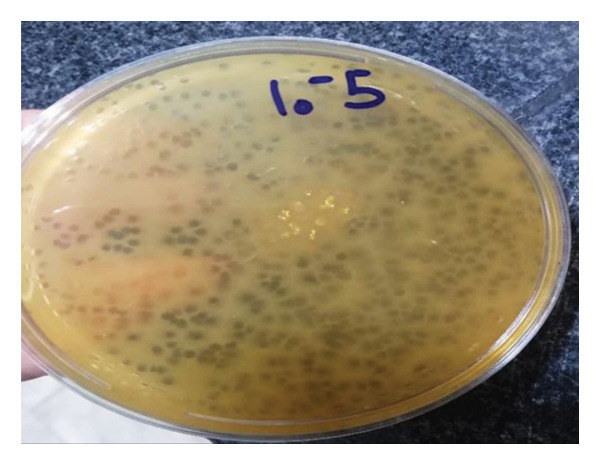
Plaque morphology and titer of the isolated ETEC‐specific bacteriophage.

The isolated phage was capable of producing small and clear plaques. The double‐layer agar method was performed to determine the bacteriophage titer. The number of ETEC phage was determined to be 2.7 × 10^9^ PFU/mL (Figure 3).

### 3.4. Phage Host Range

The specificity assessment of the phage revealed that the isolated phage exclusively targets the examined pathotype and does not induce lysis in other bacterial strains (Table 1).

**Table 1:** Phage sensitivity of tested bacterial strains determined by spot test for the ETEC phage.

Bacterial strain		ATCC	Phage sensitivity
1	*Klebsiella pneumoniae*	700603	—
2	*Escherichia coli*	25922	—
3	*Staphylococcus aureus*	225923	—
4	*Shigella flexneri*	12022	—
5	*Salmonella enterica* serovar *Typhimurium*	14028	—
6	*Enterococcus faecalis*	29212	—
7	*Enterobacter aerogenes*	13048	—

### 3.5. Phage Morphology

**Figure 4:**
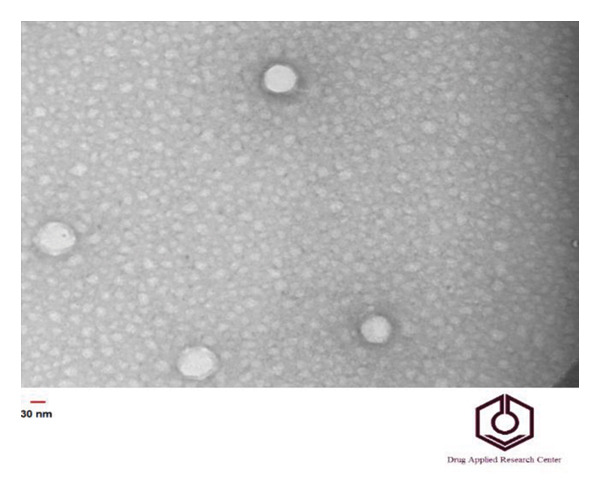
Transmission electron microscopy (TEM) image of the isolated ETEC bacteriophage showing an icosahedral head and short noncontractile tail characteristic of *Autographiviridae*.

TEM analysis showed that the bacteriophage consisted of an icosahedral head (135 ± 5 nm) and a noncontractile tail (19 ± 2 nm) belonging to the Autographiviridae family, which is shown in Figure 4.

### 3.6. One‐Step Growth

**Figure 5:**
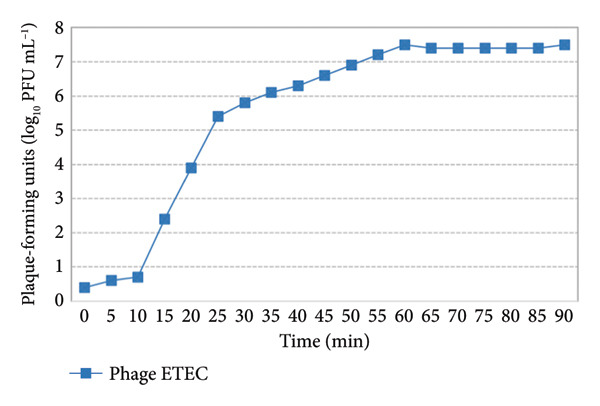
One‐step growth curve of the isolated bacteriophage infecting *enterotoxigenic Escherichia coli* (ETEC).

Analysis of the one‐step growth curve showed that the latent period of phage was from 0 to 10 min. Also, the burst size (the number of phage released from an infected bacterial cell) of the phage was 93 PFU/infected cell, indicating a highly efficient replication cycle for this phage (Figure 5).

### 3.7. Thermal and pH Stability

**Figure 6:**
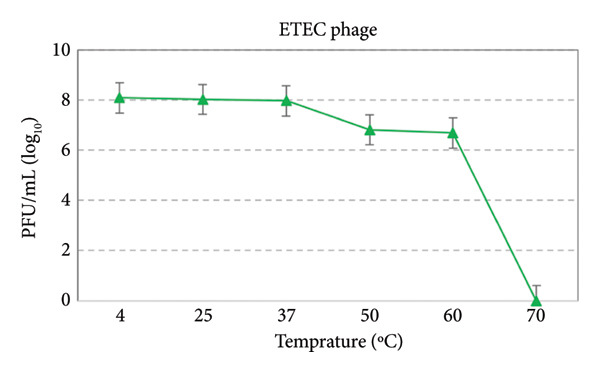
Temperature stability test curve of ETEC phage.

**Figure 7:**
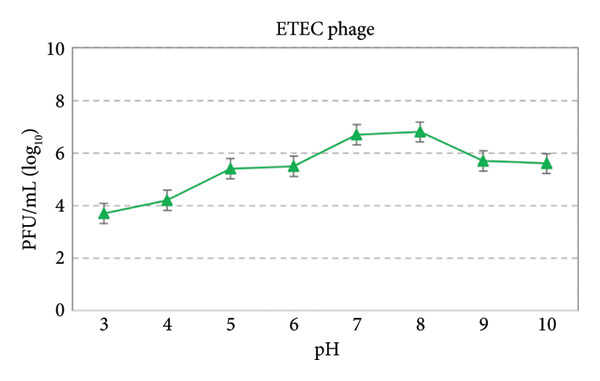
Acid and alkaline tolerance test curve of ETEC phage.

At temperatures of 4°C, 25°C, 37°C, 50°C, and 60°C, the phage remained highly stable with minimal reduction in PFU/mL. This indicates that the phage retains its infectivity under typical environmental and physiological conditions. At 70°C, no detectable infectivity was observed, indicating complete inactivation of the phage. Examining the studied phage at different pH values showed that it is unstable in high acid and alkaline conditions and performed best at pH values of 7 and 8 **(**Figures 6 and 7).

### 3.8. Antibacterial Activity of Phage

**Figure 8:**
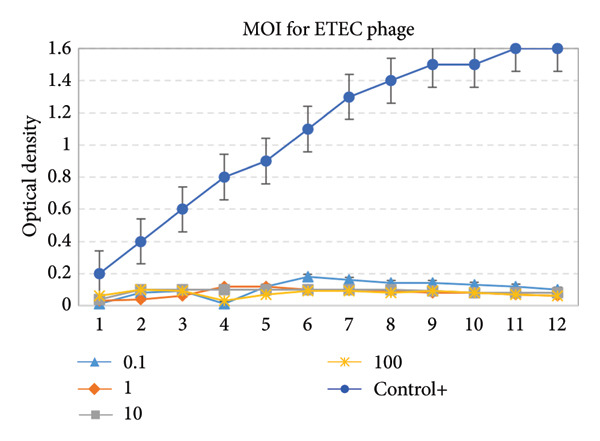
Effect of different multiplicities of infection (MOI) on bacterial growth inhibition by the ETEC phage.

At MOI values of 0.1 and 1, the optical density of the bacterial culture showed minimal reduction compared to the control group. At an MOI of 10, significant bacterial lysis was observed, as indicated by a sharp decrease in optical density. At an MOI of 100, completed bacterial lysis occurred rapidly. Therefore, based on these results, all four MOI values exhibit nearly identical functionality. This phage demonstrates a relatively low dependence on dosage and performs effectively in terms of functional efficacy (Figure 8).

## 4. Discussion

Avian pathogenic *Escherichia coli* (APEC) is a major causative agent of colibacillosis in poultry, leading to severe extraintestinal infections and significant economic losses in the poultry industry worldwide [23]. Despite over a century since its first identification, colibacillosis remains one of the most persistent endemic diseases affecting poultry production. Among the different pathotypes, ETEC is also recognized as an important foodborne pathogen, capable of causing gastroenteritis in both animals and humans. Furthermore, feces contaminate environmental reservoirs. Consequently, humans are at risk of infection through direct contact with carrier animals or the consumption of contaminated water and food [24, 25]. Antibiotic therapy is the primary treatment for colibacillosis. Nevertheless, the treatment of this condition has been significantly impeded by the increased prevalence of multidrug resistance and a variety of virulence factors, including flagella, somatic O antigens, and capsules. Conversely, the ability to form biofilms further enhances this bacterium’s resistance to antibiotics. Due to the spread of drug‐resistant pathogens, it poses a serious risk of complications and mortality in humans worldwide [16]. The results of this study revealed that the ETEC strain isolated from poultry samples exhibited high levels of resistance to commonly used veterinary antibiotics. This extensive resistance pattern likely reflects the selective pressure caused by the overuse or misuse of antibiotics in the poultry industry, leading to the emergence of MDR strains. Antimicrobial resistance in ETEC strains not only complicates treatment strategies but also poses a risk of transferring resistance genes to other pathogenic bacteria. Also, the high biofilm‐forming capacity of this ETEC strain may be closely associated with its antimicrobial resistance, contributing to treatment failure and persistent infections. In a study conducted by Goudarztalejerdi et al., broiler‐derived avian *E. coli* isolates in Iran were found to frequently exhibit both antibiotic resistance and strong biofilm‐forming ability, which may facilitate persistent infections and increase transmission risk. These findings are in line with the results of our study [26]. In this regard, research aimed at exploring and developing alternative treatments for diseases caused by this bacterium is of paramount importance. In recent years, there has been a resurgence of interest in bacteriophages as potential antimicrobial agents. This study is centered on the isolation and characterization of a specific bacteriophage targeting enterotoxigenic *Escherichia coli* (ETEC). The morphology of isolated *Escherichia coli* phage belonged to the Autographiviridae family. Additionally, this study examined the tolerance of *Escherichia coli* pathotype to varied pH levels, indicating that the phages demonstrated optimal performance at pH values of 7 and 8. Bacteriophages are usually less stable in acidic environments due to the denaturation of their proteins. Kim et al. [7] investigated the stability of a lytic phage against enterotoxigenic *Escherichia coli* at different pH levels. The results showed that the phage under study produced notable plaques over a wide pH range of 3–11. In another study conducted by Jamal et al. [27] to evaluate the stability of an *Escherichia coli* bacteriophage resistant to pH variations within the range of 3–11, it was demonstrated that the studied phage remained stable across all tested pH levels. However, the optimal performance was observed within the pH range of 7–9. The results of these investigations indicated the stability of the bacteriophage throughout a wide range of pH levels, which agrees with the findings of the present study.

The one‐step growth curve data showed that the enterotoxigenic *Escherichia coli* phage had a latent period of 10 min and a burst size of 93 PFU/cell, which is smaller than CEV1 (150 PFU/cell) [28] and E212 (125 PFU/cell) [29] but larger than JS09 (79 PFU/cell) [30]. The latent period was the minimum amount of time needed for bacteriophage particles to adsorb and release their offspring from host cells that were infected. A shorter latent period indicated that phages might infect host bacteria faster. A smaller burst size indicated the phage’s lower effect on infection. To determine the effective phage titer, we employed the MOI determination technique, conducting the assay based on MOI values of 0.1, 1, 10, and 100. For the ETEC phage, all four MOI values demonstrated nearly identical performance, indicating that this phage is not highly dose‐dependent and exhibits strong functional efficacy. A study conducted by Hu et al. [31] revealed that the most effective MOI for controlling the strains was MOI = 100. In another study by Wang et al. [32], which investigated a specific bacteriophage effective not only against *Escherichia coli* but also against *Salmonella enteritidis* and *Salmonella typhimurium*, the optimal MOI for controlling the strains was reported to be MOI = 1. In a further study by Zhou et al. [33], which aimed to control EHEC O157:H7 and enterotoxigenic *Escherichia coli* strains in food products, it was demonstrated that the best performance for controlling the strains was achieved at MOI = 0.1, 1, and 10. The results of these studies align with the findings of the current study.

The other studies have highlighted the potential of bacteriophages as effective antibacterial agents against MDR bacteria. High bactericidal activity was observed by Karami et al. in their evaluation of two novel phages against MDR avian pathogenic *E. coli*, indicating that phages can be used as safe biocontrol agents [34]. Moreno et al. demonstrated that a phage cocktail effectively reduced *E. coli* populations in poultry, illustrating the practical potential of phage therapy in industrial settings [35]. Taken together, these findings suggest that specific phages, such as the one isolated in this study, may provide a targeted approach for controlling MDR *E. coli* in poultry.

Although the findings of this study are promising, they should be considered preliminary. Further investigations involving larger sample sizes, diverse bacterial strains, and in vivo models are required to confirm the efficacy, stability, and safety of the isolated phage under field conditions. Moreover, whole‐genome sequencing (WGS) is recommended to comprehensively characterize the phage genome, confirm the absence of virulence or lysogenic genes, and better elucidate its therapeutic potential. This step is planned for the next phase of our research to provide a detailed genomic characterization and ensure the safety of the phage for future therapeutic applications. Overall, these results highlight the potential of bacteriophages as an adjunct or alternative strategy to antibiotics for the control of ETEC infections, particularly in poultry production systems.

## 5. Conclusions

Due to the improper and unevaluated use of antibiotics in veterinary medicine, the residual effect on livestock, which is transmitted to humans through the food chain and increases antibiotic resistance, has made treatment a major challenge. The high overall prevalence of colibacillosis in livestock farms and the resulting mortality and economic losses are very worrying. Accordingly, it is necessary to provide alternative treatments. It is worth noting that phage therapy has a history of 100 years and, given the very high diversity, identification, isolation, and determination of laboratory characteristics of these biological agents, is essential before clinical use. In this study, hospital wastewater was used as the best place to isolate specific phages. The characteristics of the phage extracted in this study were tolerance to strong acidic and alkaline conditions and stability at relatively high temperatures, which make it suitable for clinical use. Another important characteristic of the extracted phage is a short latent period of 10 min and a high burst size of 93 PFU per cell, indicating its strong lytic activity and potential efficacy as a therapeutic agent against ETEC infections. The specificity of the phage studied to the host pathogen species without effect on other pathogen species was another suitable characteristic of this phage.

## Disclosure

All authors read and approved the final manuscript.

## Conflicts of Interest

The authors declare no conflicts of interest.

## Author Contributions

Conceptualization and supervision: Dr. Fatemeh Fardsanei. Project administration: Dr. Farhad Nikkhahi. Investigation and methodology: Arina Sasoon. Statistical analysis: Dr. Amir Javadi. Writing—original draft: Niloofar Kiaheyrati. Investigation: Amir Peymani. Revised the manuscript: Anita Fard Sanei.

Arina Sasoon, Farhad Nikkhahi, and Niloofar Kiaheyrati contributed equally to this work.

## Funding

No funding was obtained for this manuscript.

## Data Availability

The data that support the findings of this study are available from the corresponding author upon reasonable request.
